# Intermammary Pilonidal Sinus

**DOI:** 10.4103/0974-7753.77526

**Published:** 2010

**Authors:** Anil Sunkara, DD Wagh, Sameer Harode

**Affiliations:** Department of Surgery, Jawaharlal Nehru Medical College, Sawangi (Meghe), Wardha - 442 004, Maharashtra, India

Sir,

Pilonidal sinus is a blind-end tract lined with granulation tissue, which leads to a cystic cavity lined with epithelial tissue. As the name suggests, these are hair containing abscesses, usually found in the sacrococcygeal region. However, they may also occasionally occur in the axilla, groin, interdigital web, umbilicus, nose, intermammary areas, suprapubic area, clitoris, prepuce, penis, occiput or on the feet. The hair forms small cavities or pits, which are in truth, enlarged hair follicles, which go on to become sinuses. Bacteria and debris enter this sterile area, producing local inflammation and formation of pus-filled abscesses. In chronic condition, the sinus becomes an open cavity, constantly draining small amounts of fluid. It is observed most commonly in people aged 15 to 30 years, occurring after puberty when sex hormones are known to affect the pilosebaceous gland and change healthy body hair growth. The onset of Pilonidal disease is rare in people older than 40 years. We report a rare case of Intermammary Pilonidal sinus in a fatty, hirsute female.

**Figure 1:**
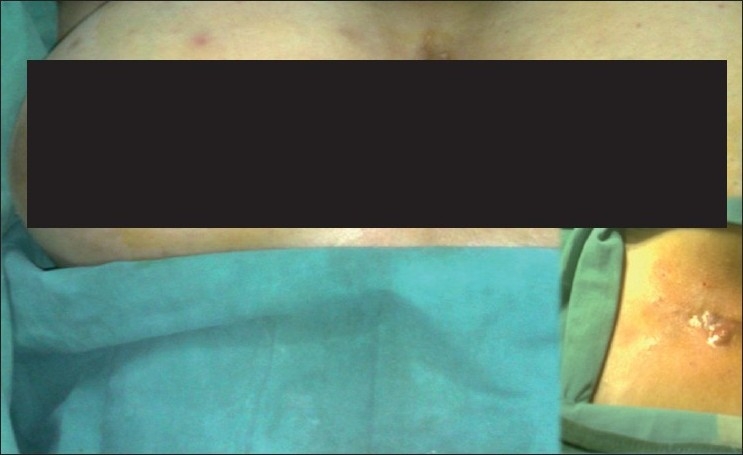
Pilonidal sinus of the intermammary region, inset showing close view

A 16-year-old, fatty, hirsute female presented with a discharging sinus over the intermammary region since two years. She initially got treated by a dermatologist for folliculitis and later approached the surgery outpatient department as the lesion did not subside [Figure 1].

**Figure 2:**
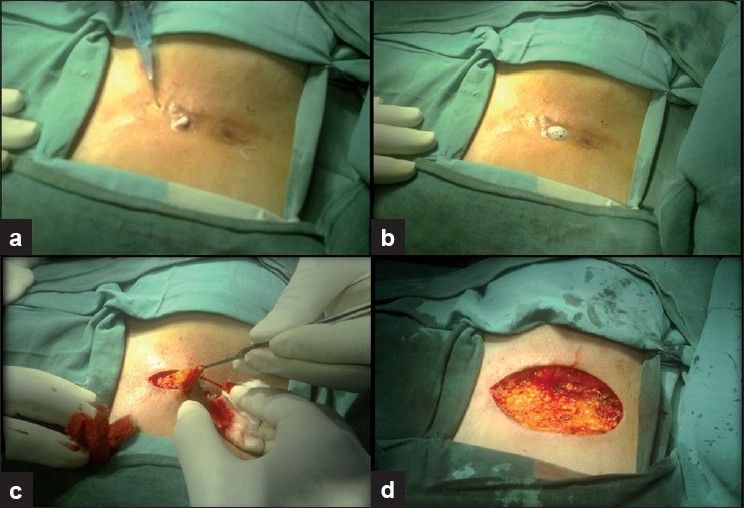
(a) Methylene blue injected in the sinus tract; (b) Sinus tracts identified; (c) Excised sinus tracts; (d) Raw surface after complete excision

**Figure 3:**
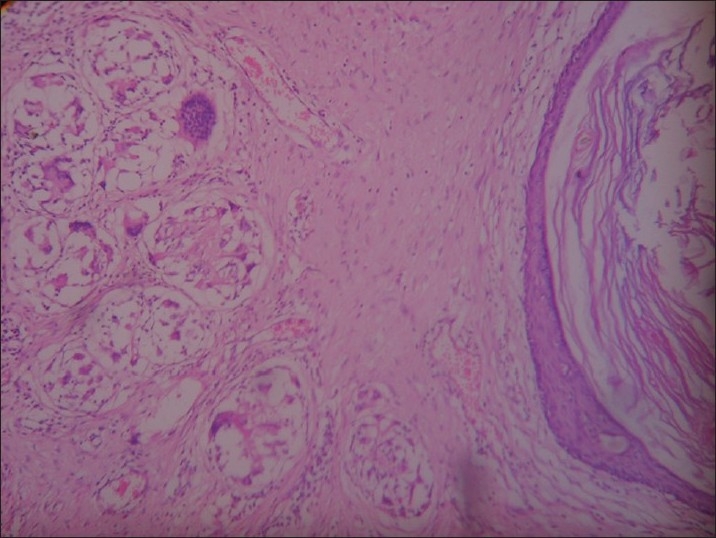
Histopathological slide showing sinus tract with chronic inflammatory cells ×10

Local examination showed multiple discharging sinus tracts with surrounding induration [Figure 1]. Complete excision of all the sinus tracts en bloc was done after confirmation of the sinus tracts by injecting methylene blue [Figure 2a–c]. Primary closure was done and drain left in, which was removed when the 24 hour collection was less than 5 ml [Figure 2d]. The histopathology showed sinus tracts with chronic inflammatory tissue, confirming the diagnosis [Figure 3]. The postoperative period was uneventful and the wound at follow-up after 3 months showed clear margins.

In 1833, Herbert Mayo described a cyst that contained hair just below the coccyx. Hodge in 1880 coined the name ‘pilonidal’ from the Latin words *pilus*, which means hair, and *nidus*, which means nest.[1,2] Pilonidal disease consists of a spectrum of entities ranging from asymptomatic hair containing cysts and sinuses to a large abscess.

The medical literature regarding the etiology of the pilonidal cyst has shifted. Initially, these cysts were believed to be congenital in nature. Pilonidal disease is now widely accepted as an acquired disorder based on the observations that congenital tracts do not contain hair and are lined by cuboidal epithelium. The recurrence of the disorder after complete excision of the disease tissue and the high incidence of chronic pilonidal sinus disease in patients who are hirsute further support an acquired theory of pathogenesis.[1,3]

A Pilonidal sinus is a blind-end tract lined with granulation tissue, which leads to a cystic cavity lined with epithelial tissue. As the name suggests, these are hair containing abscesses, usually found in the sacrococcygeal region. However, they may also occasionally occur in the axilla, groin, interdigital web, umbilicus, nose, intermammary areas, suprapubic area, clitoris, prepuce, penis, occiput or on the feet.[4,5] Intermammary pilonidal sinus disease is commonly seen in fatty females with increased distribution of hairs. After the onset of puberty, sex hormones affect the pilosebaceous glands, and, subsequently, the hair follicle becomes distended with keratin. As a result, a folliculitis is created, which produces edema and follicle occlusion. The infected follicle extends and ruptures into the subcutaneous tissue, forming a pilonidal abscess. This results in a sinus tract that leads to a deep subcutaneous cavity. The direction of the sinus tract is cephalad in 90% of the cases, which coincides with the directional growth of the hair follicle. The laterally communicating sinus is created as the pilonidal abscess spontaneously drains to the skin surface. The original sinus tract becomes an epithelialized tube. The laterally draining tract becomes a granulating sinus tract opening.[1]

The sinus is caused by the friction of the skin leading to the embedding of the hair beneath the surface. The hair forms small cavities or pits, which are in truth, enlarged hair follicles, which go on to become sinuses. Bacteria and debris enter this sterile area, producing local inflammation and formation of pus-filled abscesses. In chronic condition, the sinus becomes an open cavity, constantly draining small amounts of fluid.[6,7]

Although intermammary pilonidal disease may manifest as an abscess, pilonidal sinus, recurrent or chronic pilonidal sinus, the most common manifestation of pilonidal disease is a painful fluctuant mass. Initially, 50% of patients present with a pilonidal abscess in cephalad direction to the hair follicle and/or sinus infection. Pain and purulent discharge from the sinus tract are present 70-80% of the time and are the two most frequently described symptoms. In the early stages prior to the development of an abscess, only a cellulitis or folliculitis is present. The abscess is formed when a folliculitis expands into the subcutaneous tissue or when a preexisting foreign body granuloma becomes infected. The subcutaneous cavity and laterally oriented secondary sinus tract openings are lined with granulation tissue, whereas only the midline natal cleft pit sinus is lined by epithelium. The diagnosis of a pilonidal sinus can be made by identifying the epithelialized follicle opening, which can be palpated as an area of deep induration beneath the skin.[6,7]

Treatment for symptomatic intermammary pilonidal sinus involves surgery to incise and drain the abscess. The surgery can be either wide excision and healing by secondary intention (longer healing time, low chance of recurrence), excision and primary closure by sutures (quicker healing, risk of recurrence), or plastic surgery technique (for recurring and/or extensive sinus).[8] The other procedures evolving are with topical natural polyphenols/laser epilation.[9,10]
